# Supplementary material for: Contextual and individual factors associated with self-reported tooth loss among adults and elderly residents in rural riverside areas: A cross-sectional household-based survey
Source: PLoS One. 2022 Nov 22;17(11):e0277845. doi: 10.1371/journal.pone.0277845 (PMC9681076; doi:10.1371/journal.pone.0277845)
Supplement: Attachment — Submitted filename: Response to Reviewers_R2.docx [file pone.0277845.s002.docx]

We thank the reviewers for their comments and/or suggestions, which helped to improve the manuscript. We have numbered their comments to organize the responses. We shall deal with each comment here:

Reviewer #1

The authors work hard in the manuscript and letter to reply to the comments. Some points yet need to be discussed.

1) In the objective, I would like to read a shorter objective as: Objective: To identify contextual and individual factors with self-reported tooth loss among adults and elderly people living in rural riverside areas.

Answer: The study objective was rewritten, as suggested by the reviewer.

Abstract, Objective

Removed: “To identify the association between socioeconomic and service-related contextual factors and socioeconomic, demographic, behavioral and self-perceived oral health individual factors with self-reported tooth loss among adults and elderly people living in rural riverside areas.”

Added: “To identify contextual and individual factors associated with self-reported tooth loss among adults and elderly people living in rural riverside areas.”

Introduction, last paragraph

Removed: “Thus, the aim of the study was to evaluate the association of socioeconomic and service-related contextual factors and socioeconomic, demographic, behavioral and self-perceived oral health individual factors with self-reported tooth loss among adults and elderly people living in rural riverside areas.”

Added: “Thus, the aim of the study was to evaluate the association of contextual and individual factors with self-reported tooth loss among adults and elderly people living in rural riverside areas.”

2) I also emphasize that when the authors describe the health services in North Region, it is important to contextualize the problem is not to be dependent of public health service, but there is a problem of accessibility for health. There is a lack in health service include private service. Besides Brazil have a universal health service, there are difficulties to manage the financial resources (lack of resources) and also is a too big country and unequal. I think these points could be in discussion section. Please this fact clear in introduction.

Answer: Certainly, the issues raised are relevant to the understanding of access to health services in the North region. The low availability of services (public and private), added to geographic and financial barriers, and other social and organizational aspects (such as accommodation and acceptability), depicts a challenging scenario in such an unequal country. These aspects were addressed in the Introduction and in the Discussion section of the manuscript.

Introduction, 5th paragraph

Removed: “Contextual characteristics of geographic location contributes to inequalities between rural and urban areas, both in general and oral health. The urban environment can mitigate the negative aspects present in the rural environment, such as geographic barriers, socioeconomic deprivation, and limited access to health services [31-35]. Despite having a strong dependence on public healthcare services [36], the physical network of services is inadequate and health professionals are lacking in rural areas in Brazil [37]. The northern region of Brazil has the worst rates of use of health services in the country [38]. Health policies in the region lack institutional structure, continuity, and sensitivity to regional specificities. To the limited provision and organization of healthcare of services clinics are associated low income, population dispersion, the large geographic distances and other barriers to access typical of the Amazon region [39-41].”

Added: “Contextual characteristics of geographic location contributes to inequalities between rural and urban areas, both in general and oral health. The urban environment can mitigate the negative aspects present in the rural environment, such as geographic barriers, socioeconomic deprivation, and limited access to health services [31-35]. Brazil is a large and unequal country, and the northern region has the worst rates of use of health services in the country [38]. Despite having a strong dependence on public healthcare services [36], the physical network of public and private services is inadequate and health professionals are lacking in rural areas in Brazil [37]. Health policies in the region lack institutional structure, continuity, and sensitivity to regional specificities. In addition to the limited provision and organization of healthcare of services clinics, there are low income, population dispersion, the large geographic distances and other barriers to access typical of the Amazon region [39-41].”

Discussion, 3rd paragraph

Maintained: “This would contribute to a more rational use of the limited human, financial and structural resources characteristic of the northern region of Brazil and also to a greater resoluteness of health care [39-40].”

Discussion, 9th paragraph

Added: “A family with better financial resources is also more able to overcome low availability and other barriers to accessing health services [39-41,50].”

3) In methods when the authors mentioned 2019 means January 2019 until December 2019?

Answer: Information about the months has been added to the Methods section.

Methods, 1st paragraph

Removed: “A household-based cross-sectional survey was conducted in 2019 in rural riverside localities…”

Added: “A household-based cross-sectional survey was conducted from March to July 2019 in rural riverside localities…”

4) I did not understand the sample calculation and sample selection. Why was the sample calculation 751 and 340 were estimated to be visited? Maybe is better make clear that the mean of people in each house is 2, and in order to reach the sample size that is calculated for individuals, 340 houses were selected.

Answer: As suggested by the reviewer, the explanation was added in the paragraph of the sample calculation, in order to make it clearer.

Methods, 2nd paragraph

Removed: “Considering the probability of finding individuals from each group within households, a number of 340 households were estimated to be visited in the study…”

Added: “Considering the probability of finding individuals from each group within households (average of 2.215 adults per household), a number of 340 households were estimated to be visited in the study…”

5) In the methods it is important to make clear the way the variables were analyzed, if count or noun variables and the category to avoid confusion.

Answer: The dependent variable, number of missing teeth, was evaluated as a count outcome, as also described in the Methods section. Information on independent variables was added to the text, as suggested by the reviewer.

Methods, 5th and 6th paragraphs

Removed: Independent variables were selected according to the theoretical background presented on the determinants of tooth loss. Contextual variables included for each territory were per capita income, average household income, poverty (family income less than US$ 5.50 per day) and extreme poverty (family income less than US$ 1.90 per day) rates, Gini index of per capita income and household income. These variables were calculated based on information from the households evaluated in the territories. Characteristic of primary healthcare was also assessed at a contextual level, including: 1) locations covered by fluvial health teams (FHT) and CHW, with health unit in the community, 2) locations covered by FHT and CHW, without health unit in the community, 3) locations only covered by CHW, and 4) locations covered by riverside health teams (RHT).

Individual characteristics were sex, age, race/skin color, household income, registration in the Bolsa Família program (Brazilian conditional cash transfer program for low-income families), occupation (yes/no), monthly sugar consumption of family, time since last dental appointment, dental pain over the past six months (yes/no) and satisfaction with teeth/oral health. Time since last dental appointment was evaluated according to the categories: over the past 12 months, more than 1 year up to 2 years ago, more than 2 years up to 3 years ago, more than 3 years ago, and never have been to the dentist. Satisfaction was assessed using a 5-point Likert scale, ranging from very satisfied to very dissatisfied. Sugar consumption was evaluated through the availability inferred by the frequency of monthly purchases of sugary foods at home.”

Added: “Independent variables were selected according to the theoretical background presented on the determinants of tooth loss. Contextual variables included for each territory were per capita income, average household income, poverty (family income less than US$ 5.50 per day) and extreme poverty (family income less than US$ 1.90 per day) rates, Gini index of per capita income and household income. These continuous numeric variables were calculated based on information from the households evaluated in the territories. Characteristic of primary healthcare was also assessed at a contextual level using a nominal categorical variable, including: 1) locations covered by fluvial health teams (FHT) and CHW, with health unit in the community, 2) locations covered by FHT and CHW, without health unit in the community, 3) locations only covered by CHW, and 4) locations covered by riverside health teams (RHT).

Individual characteristics were sex (male/female), age (discrete numeric variable), race/skin color (white/black/brown/Asiatic/indigenous), household income (continuous numeric variable), registration (yes/no) in the Bolsa Família program (Brazilian conditional cash transfer program for low-income families), occupation (yes/no), monthly sugar consumption of family, time since last dental appointment, dental pain over the past six months (yes/no) and satisfaction with teeth/oral health. Time since last dental appointment was evaluated according to the categories: over the past 12 months, more than 1 year up to 2 years ago, more than 2 years up to 3 years ago, more than 3 years ago, and never have been to the dentist. Satisfaction was assessed using a 5-point Likert scale, ranging from very satisfied to very dissatisfied. Sugar consumption was evaluated in kilograms (continuous numeric variable) through the availability inferred by the frequency of monthly purchases of sugary foods at home.”

6) Were the examiners calibrated?
Answer: As the survey was conducted with closed-ended interview questions, the answers to which were reported by the participants, the examiners were trained, and a pilot study was carried out in rural communities not involved in the main study. No clinical measurement was performed in the study.

Methods, 4th paragraph

“After 40 hours of theoretical and practical training, a pilot study was conducted in two rural communities, from territories other than those included in the main study.”

7) I understand the explanation for the variable missing teeth, but it will be interesting to observe which category is more prevalent in this population, non-functional dentition? More than 8 teeth? Edentulism? If the sum of the categories is 100% it would be better to comprehend the result and the distribution of the interest variable. If they are not mutually exclusive you cannot observe the real distribution.

Answer: The number of missing teeth, a discrete numerical variable (count outcome) was the main outcome of interest for the study. The other dichotomous outcomes (edentulism, severe tooth loss and non-functional dentition) were also described to portray the rural riverine population tooth loss scenario more comprehensively. As described in the Methods section and noticed by the reviewer, these categories are not mutually exclusive, and for this reason they were not presented in this way. An edentulous individual certainly did not also have a functional dentition. These classifications of tooth loss have been widely used in studies on tooth loss and were reproduced in the same way in the present study. With mutually exclusive categories (if applicable), of the 27.4% who had non-functional dentition, excluding 12.1% of edentulous, the remaining 15.3% would be divided into severe tooth loss (7.5%) and non-functional dentition (7.8%). We emphasize that, even if not presented in the text, these values can still be calculated when evaluating the difference between the frequencies of the not mutually exclusive categories, in a sequential way (e.g., 27.4-19.6=7.8%, 19.6-12.1=7.5%).

Parker ML, Thornton-Evans G, Wei L, Griffin SO. Prevalence of and Changes in Tooth Loss Among Adults Aged ≥50 Years with Selected Chronic Conditions - United States, 1999-2004 and 2011-2016. MMWR Morb Mortal Wkly Rep. 2020 May 29;69(21): 641-646.

Ribeiro CG, Cascaes AM, Silva AE, Seerig LM, Nascimento GG, Demarco FF. Edentulism, Severe Tooth Loss and Lack of Functional Dentition in Elders: A Study in Southern Brazil. Braz Dent J. 2016 May-Jun;27(3): 345-52.

Peres MA, Lalloo R. Tooth loss, denture wearing and implants: Findings from the National Study of Adult Oral Health 2017-18. Aust Dent J. 2020 Jun;65 Suppl 1: S23-S31.

Kassebaum NJ, Bernabé E, Dahiya M, Bhandari B, Murray CJ, Marcenes W. Global Burden of Severe Tooth Loss: A Systematic Review and Meta-analysis. J Dent Res. 2014 Jul;93(7 Suppl): 20S-28S.

8) In discussion, what is more than a quarter? 25%?
Answer: 27.4% of adults had non-functional dentition. The intention was not to repeat the numerical value already presented in the Results section, but to draw attention to its expressiveness within the sample. In view of the reviewer's question, the sentence was rewritten.

Discussion, 2nd paragraph

Removed: “…and more than a quarter presented non-functional dentition.”

Added: “…and approximately 27% presented non-functional dentition.”

9) The conclusion needs to be rewritten in order to be based on the main results and answer the objective. What are the associated factors of tooth loos in individual and contextual factors?

Answer: The authors agree with the reviewer. When the last paragraph of the discussion was reorganized to become the conclusion during the review process, it ended up that way. So, the conclusion was now rewritten based on the study objectives and the main results, as well as pointing out at the end some implications of the study findings, as also suggested throughout the review process.

Conclusions

Added: “The study population presented a high number of self-reported missing teeth. The presence of RHT was a contextual protective factor for tooth loss. As for individual factors, older age, white race/skin color, lower family income, non-inclusion in the Brazilian cash transfer program, higher sugar consumption, dental pain, worse self-perceived oral health and not having had a dental appointment over the past year were associated with a higher number of missing teeth.”

Reviewer #2

The answers were answered satisfactorily. The study has methodological quality and is relevant above all because it is in a poorly studied and culturally differentiated population. The results can contribute to the planning of specific policies for this population. Congratulations on the work.

Answer: The authors are grateful for the reviewer's suggestions, which contributed to improving the manuscript.
